# Heme oxygenase 1: a novel oncogene in multiple gynecological cancers

**DOI:** 10.7150/ijbs.61073

**Published:** 2021-06-01

**Authors:** Jia-Jing Lu, Ayitila Abudukeyoumu, Xing Zhang, Li-Bing Liu, Ming-Qing Li, Feng Xie

**Affiliations:** 1 Medical Center of Diagnosis and Treatment for Cervical Diseases, Obstetrics and Gynecology Hospital of Fudan University, Shanghai 200011, People's Republic of China.; 2 Laboratory for Reproductive Immunology, NHC Key Lab of Reproduction Regulation (Shanghai Institute of Planned Parenthood Research), Hospital of Obstetrics and Gynecology, Fudan University, Shanghai 200080, People's Republic of China.; 3 Department of Gynecology, Changzhou No.2 People's Hospital, affiliated with Nanjing Medical University, Changzhou, Jiangsu Province, 213003, People's Republic of China.; 4 Shanghai Key Laboratory of Female Reproductive Endocrine Related Diseases, Hospital of Obstetrics and Gynecology, Fudan University, Shanghai 200080, People's Republic of China.

**Keywords:** Heme Oxygenase-1, proliferation, metastasis, cancer, oxidative stress

## Abstract

Heme oxygenase 1 (HO-1), also known as heat shock protein 32 (HSP32), is a stress-inducible enzyme. In the past, it was believed to participate in maintaining cell homeostasis, reducing oxidative stress damage and exerting anti-apoptotic effects. When exposed to noxious stimulation, the expression of HO-1 in the body will increase, antagonizing these oxidative stresses and protecting our bodies. Recently, many studies showed that HO-1 was also highly-expressed in multiple gynecological cancers (such as ovarian cancer, cervical cancer and endometrial cancer), suggesting that it should be closely related to cell proliferation, metastasis, immune regulation and angiogenesis as an oncogene. This review summarizes the different effects of HO-1 under normal and diseased conditions with a brief discussion of its implications on the diagnosis and treatment of gynecological cancers, aiming to provide a new clue for prevention and treatment of diseases.

## Introduction

The most important component of red blood cell is hemoglobin, which is composed of globin and heme 1. Heme is a large complex, containing iron and protoporphyrin IX 2-4. It is a cellular oxidant, participating in the formation of oxidative free radicals and leading to oxidative injury 5. Noxious stimulation leads to the increase of heme oxygenase 1 (HO-1) for antagonizing these oxidative stresses and protecting our bodies 6. Therefore, the hypothesis that HO-1 may be used as a targeted gene in tumor treatment has attracted more and more attentions. It is one of the most widely distributed antioxidative enzymes in the body and is the rate-limiting enzyme of heme metabolism 7.

Among all these malignant tumours, gynecological cancers is a specific type of fatal disease which only happens to women, and can seriously threaten the lives and health of women around the world. In recent years, many studies have confirmed that HO-1 is highly-expressed in a variety of gynecological tumors, such as ovarian cancer, cervical cancer and endometrial cancer. The elevated level of HO-1 and the deviation of its dynamic trend from the baseline may be a signal of disease alert 8-11. Studies have also shown that as a novel oncogene, HO-1 is closely related to tumor proliferation and metastasis, and may become a potential marker for predicting the prognosis of gynecological tumors 9. Furthermore, HO-1 itself is also expected to become a target for tumor treatment. HO-1 inhibitors such as zinc protoporphyrin (ZnPP) have obtained certain efficacy in clinical work 12. Therefore, this review summarizes the expression, regulation, roles and treatment values of HO-1 in gynecological tumors.

## HO-1 and heme metabolism

### HO family and HO-1

In the long course of evolution, cells have developed a set of mechanisms against oxidative stress, and heme/HO system is one of the most important anti-oxidant mechanisms. Since Tenhunen and his colleagues first described the mechanism of heme catabolism in 1968 13, people have gradually conducted more and more studies on the HO family. It is an unique type of cell protection enzyme located in the endoplasmic reticulum, and participate in the metabolism of heme 14.

From algae to humans, HO is ubiquitous and highly-conserved, suggesting that it may play an indispensable role in cells. We usually divide HO into three categories 15. As shown in **Table 1**, HO-1 is a 32 kD stress-inducible enzyme 16. As it can be induced by high temperature, so it is also known as heat shock protein 32 (HSP-32) 13, 17. High-expression of HO-1 can be observed in liver, spleen, bone marrow and senescent erythrocytes 18, 19, and its main function is to degrade heme into biliverdin (BV), carbon monoxide (CO) and ferrous ion (Fe^2+^) 20, 21. Under the action of biliverdin reductive (BVR), BV will be further processed into bilirubin (BR) 22, 23. By regulating intracellular levels of heme and heme metabolites, HO-1 participates in maintaining cell homeostasis, reducing oxidative damage, regulating cell proliferation and apoptosis 24. HO-2 is a 36 kD constitutive enzyme, which is related to the nerve creed effect of carbon monoxide, it also participates in hemoglobin degradation 25. High levels of HO-2 can be found in brain tissue, retina and testis 26. Many scholars speculate that the presence of HO-2 in the testis may play a crucial role in the male reproductive system 27, but this hypothesis has not been confirmed. What has been verified is that HO-2 can protect neurons against ischemia/reperfusion injury. HO-3 is widely distributed but has weak activity, which can promote the combination of heme and HO 28. Sometimes it is viewed as a pseudogene processed from HO-2 transcription 26. Among different HO isoforms, HO-1 seems to be the most valuable because its expression level can be induced under various pathophysiological conditions 24.

### Metabolites related to HO-1

#### Carbon monoxide

**Figure 1:**
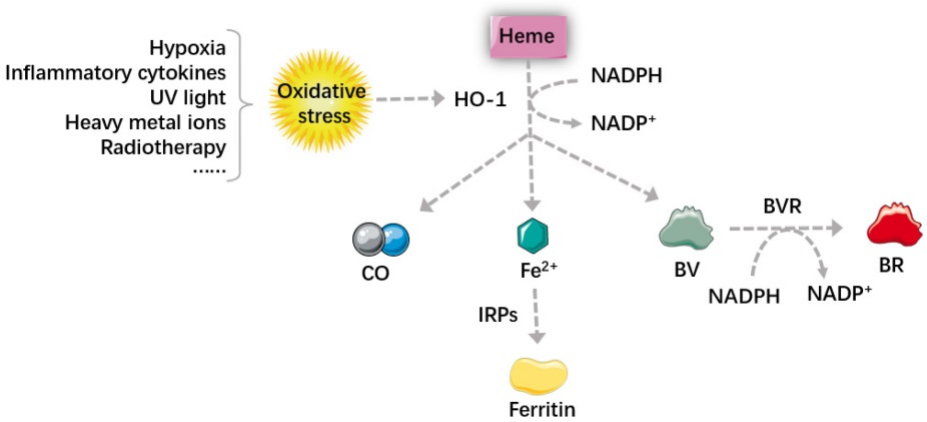
** Heme metabolism and its main metabolites.** Oxidative stress increases the level of HO-1, which can degrade heme into BV, BR, CO and Fe^2+^. Through regulating the autonomic nervous system, CO can act directly on the blood vessels to cause vasodilation. Large amount of Fe^2+^ generated by heme metabolism can dissociate IRPs from mRNA and promote the translation of ferritin, which increases the anti-injury ability of cells and makes the tumors more sensitive to therapy. BVR is an NADPH-dependent enzyme, and it can reduce BV into BR. BV and BR then work together to scavenge excessive ROS and inhibit the peroxidation of lipid and protein. In summary, by regulating intracellular levels of heme and heme metabolites, HO-1 participates in maintaining cell homeostasis, reducing oxidative damage, regulating cell proliferation and apoptosis. HO-1: Heme oxygenase 1; BV: biliverdin; BR: bilirubin; CO: carbon monoxide; Fe^2+^: ferrous ion; IRPs: iron regulatory proteins; BVR: biliverdin reductive; ROS: reactive oxygen species.

As depicted in **Figure 1**, carbon monoxide (CO) is a gaseous product, most CO in our bodies comes from heme metabolism, it is an important signaling molecule 22, 29. It can act directly on the blood vessels to cause vasodilation, this effect can also be achieved through the activity of the autonomic nervous system 30, 31. Besides, CO plays a significant role in anti-apoptotic effects, this can be synergistic with other anti-apoptotic system 32, 33, by regulating the Mitogen-activated protein kinase (MAPK) signal pathway and inhibiting the activated mononuclear macrophage system. Exogenous CO has also this effect by up-regulating the expression of HO-1. Additionally, CO also participates is involved in many other physiological and pathological processes, for example, anti-inflammation, anti-proliferation 16, 34, inhibition of platelet aggregation 22, 35 and neurotransmission 36.

#### Biliverdin and bilirubin

BVR is an NADPH-dependent enzyme, which can reduce biliverdin (BV) to bilirubin (BR) in the presence of NADPH. It is a dual-specific protein kinase that phosphorylates serine and tyrosine, therefore it's a key enzyme for protein phosphorylation. Apart from working as reductase and dual-specific protein kinase, BVR can also regulate tumor cells by acting as a basic leucine zipper (bZIP) transcription factor 37, 38.

The anti-apoptotic effect of BV and BR is achieved by promoting anti-apoptotic protein bcl-2 and inhibiting pro-apoptotic protein Bax 39. BR is also an important antioxidant in our bodies 40, it can inhibit the peroxidation of lipid and protein by scavenging excessive reactive oxygen species (ROS) 20, 41. Oxidative stress is caused by the imbalance between cellular oxidants and antioxidants, ROS is a major oxidant in our bodies 42, 43, which can cause protein denaturation, genetic instability, and promote tumorigenesis 43. It is usually generated by oxygen metabolism, but noxious stimulation can also cause an increase in ROS 44.

#### Ferrous ion

Fe^2+^ is cytotoxic because it can interact with cellular oxidants to generate ROS, promoting oxidation and inflammation. However, Fe^2+^ generated by heme metabolism can increase the expression of ferritin, which turns to prevent inflammation. In other words, when HO-1 is highly expressed, the level of Fe^2+^ increases, iron regulatory proteins (IRPs) can be dissociated from mRNA, which promotes ferritin translation. Ferritin increases the anti-injury ability of cells and makes the tumors more sensitive to oxidative stress, realizing the anti-tumor effect of HO-1 in iron metabolism. However, some studies pointed out that many diseases, such as Alzheimer, patients' lipid peroxidation may be related to iron accumulation. The amount of iron and ROS is the determinative momentum for the role of HO-1, excessive iron and ROS can turn HO-1 from a cell protector to a perpetrator, causing DNA damage, gene mutations and even cell death 45, 46. Ferroptosis is a newly-identified non-programmed cell death, which is characterized by iron-overload and lipid peroxidation 47, 48. Therefore, some researchers proposed that it can be used as a new strategy in many diseases, especially in cancer therapy.

## The role of HO-1 in different tumors

Oxidative stress is caused by the imbalance between oxidation and antioxidant system in our bodies 49. Common noxious stimuli include hypoxia, inflammatory cytokines, ultraviolet light, heavy metal ions, radiotherapy and so on 24, 47. These stimuli changes the body's homeostasis, which triggers the activation of various signaling pathways, leading to disease progression. In normal cells, HO-1 acts as a cytoprotective agent, it can fight against oxidative injuries and regulate inflammatory response 50, 51. In ischemic diseases, HO-1 has been be used for the treatment of glucocorticoid-related osteoporosis and osteonecrosis 52. Noxious stimuli actives upstream signal kinases, promoting the binding of DNA and transcription factors, which results in high expression of HO-1 and providing protection for normal cells 50, 53.

Nevertheless, the biological effect of HO-1 seems to be tissue-specific. In some cancer cells, HO-1 plays the role of survival factor 29. Overexpression of HO-1 promotes tumor progression in turn 23, 54. It can facilitate angiogenesis and prevent tumor cells from apoptosis, leading to its survival and progression 55. Researches suggest that heme can induce a stress-inducible protein Sestrin2 (SESN2), which is a protective mechanism to antagonize oxidative stress and colon tumor growth. However, high level of SESN2 can promote tumorigenesis 56. Another high-risk factor of colorectal carcer is the consumption of red meat. Heme iron is the main component of red meat, which may be the cause of excessive colonic proliferation and carcinogenesis 57. Antioxidants can stimulate lung cancer metastasis by reducing heme levels and stabilizing the transcription factor BTB and CNC homology 1 (BACH1) 58. Overexpression of HO-1 induces the expression of Cyclin Dependent Kinase 4 (CDK4) and promotes the occurrence of liver cancer; however, a feedback loop may exist between IL-6 and HO-1, thus HO-1 can be induced as an antitumor gene through the IL-6/JAK/STAT3 pathways 59, 60. The application of HO-1 inhibitor zinc protoporphyrin (ZnPPIX) can greatly inhibit the proliferation of pancreatic cancer cells, while HO-1 significantly promotes cell proliferation 61. Overexpression HO-1 promotes the occurrence of melanoma and plays the role of anti-apoptosis through the B-Raf-ERK signaling pathway 62. The most common metastatic site of prostate cancer is bone, a research by Anselmino has showed that HO-1 is a pivotal modulator of bone turnover and remodeling because it can promote the growth and invasion of cancer cells both *in vivo* and *in vitro*. This may be related to the epithelial-mesenchymal transition (EMT) induction and antioxidant and antiapoptotic effects of the prostate cancer cells 63, 64.

HO-1 can increase anti-apoptotic ability, which may lead to their uncontrolled cell proliferation and even cause tumorigenesis 24. Compared with the surrounding normal tissues, therefore, the increased expression of HO-1 can be observed in tumors 11. Abnormal signaling pathway activation can lead to reduced self-adhesion ability of tumor cells, tumor angiogenesis and changes in microenvironment 65, preventing the cancer cells from apoptosis and autophagy, and even promoting their proliferation and metastasis 47. Many studies have shown that HO-1 is a crucial substance for angiogenesis 66, which can help malignant tumors continue to grow and invasion. Currently, highly-expressed HO-1 has been found in various malignant tumors such as melanoma 2, thyroid cancer 5, osteosarcoma 50, breast cancer 67, lung cancer 58, bowel cancer 56, 57, 68, renal cell cancer 69, hepatoma 59, prostate cancers 63, 64, pancreatic cancer 61, and so on.

However, the formation of tumors is not only related to the cancer cells themselves. In fact, their occurrence, growth and even metastasis are very closely related to the surrounding cells (immune/inflammatory cells, glial cells, fibroblasts, etc.) and extracellular components (cytokines, growth factors, hormones, etc.), namely the so-called tumor microenvironment (TME) 70, 71. More and more studies have shown that HO-1 can affect cancer progression through modulating TME 72. Of note, HO-1 can act as an immunomodulator that inhibits cell maturation, activation and infiltration 73-75. Myeloid-derived suppressor cells (MDSCs) are known to inhibit anti-tumor immunity, HO-1 expression in MDSCs plays a role in the suppression of alloreactive T cells 76, 77 through promoting the release of many inflammatory factors such as interleukin-10 (IL-10) 78 and tumor necrosis factor-α (TNF-α) 79, the expression of transforming growth factor β (TGF-β), intercellular adhesion molecule 1, and other fibrogenesis factors increase, activating the NF-κB/Signal Transducer and Activator of Transcription (STAT)3 signaling pathway 80, 81, and maintaining the self-renewal ability of cancer stem cells^82^. By regulating the inflammatory response and anti-tumor immunity, these immune/inflammatory cells play significant roles in TME, which can deeply affect cancer progression.

Meanwhile, as a key mediator of angiogenesis, vascular endothelial growth factor (VEGF) can form new vasculature around tumors, causing them to grow exponentially 83. HO-1 also participates in fostering angiogenesis linked to inflammation and tumor by up-regulating the expression of VEGF in macrophages. A study conducted by Gabriel et al. has showed that the ectopic expression of HO-1 can significantly increase the transcriptional activity of VEGF in prostate cancer cells 84. In addition, VEGF fosters the formation of capillary-like tubular structures in tumor tissues 85, 86, and promotes the proliferation and migration of cancer cells.

More importantly, the level of HO-1 is closely related to the clinical features and prognosis of tumors. Generally speaking, the higher the expression of HO-1, the lower the tumor differentiation, and the more active the proliferation and metastasis 9. HO-1 inhibitors can suppress cell proliferation and invasion by increasing intracellular ROS levels and inducing cell cycle arrest 5. Because HO-1 is so closely related to tumors, some people even proposed that HO-1 can also be used as one of the tumor markers. However, a study on breast cancer showed that HO-1 overexpression can reduce lung metastasis by inhibiting cell EMT and proliferation, suggesting that HO-1 is tissue-specific 87, which needs to be studied further.

HO-1 plays different roles during different stages of tumor formation. Before a tumor is formed, it can remove aging and dead cells, inhibit tumors and protect normal cells. When a tumor is formed, the activation of HO-1 enables tumor cells to gain this anti-apoptotic ability, which leads to the occurrence and proliferation of tumors 53. In that case, it has a protective effect on tumor cells instead.

## Regulation mechanism of HO-1 expression

Different HO-1 inducers activate different protein phosphorylation-dependent signaling pathways, then activate various transcription factors. MAPK is one of the most important signal kinases in HO-1 transcription, other signal kinases such as phosphatidylinositol 3-kinase (PI3K), tyrosine kinases and many protein kinases (PK) also participate in this process 24. The latest study suggests that the regulation of its enzymatic activity depends heavily upon the expression of transcriptional level 88.

Nuclear factor E2-related factor 2 (Nrf2) is a key transcription factor involving in maintaining cell redox homeostasis 89, and HO-1 is one of the most important regulatory products. It is a bZIP transcription factor 10. Under resting conditions, Nrf2 binds to kelch-like ECH-related protein 1 (keap1) and form a Keap1-Nrf2 complex 90. The complex will be degraded by ubiquitous proteasome and exist in the cytoplasm in an inactive state. However, oxidative stress induces the modification of cysteine ​​residues in Keap1, causing Nrf2 to dissociate from the complex and increase the translocation of nucleus 10. Within the nucleus, it binds to the antioxidant-responsive element (ARE) in target gene promoters and form the Nrf2-ARE signaling pathway 90, activating the transcription of its downstream target genes, such as HO-1 and NADPH quinone dehydrogenase 1 (NQO1), protecting cells from oxidative damage and participating in maintaining redox homeostasis 24, 42, 56.

The PI3K/protein kinase B (PKB, also known as AKT) signaling is one of the most critical pathways in regulating cell growth, proliferation and apoptosis. Studies have found that Nrf2 is significantly elevated in tumor cells. Inactivating PI3K/AKT pathway can significantly reduce the level of Nrf2, inhibiting tumor cell proliferation, inducing cell apoptosis, and improving the sensitivity of tumor cells to treatment 91. In addition, MAPK signaling pathways can also regulate the activity of Nrf2.

Nuclear factor κB (NF-κB) and Bach1 also play a key role in the occurrence and development of cancer 92, and is considered to be a target for the cancer therapy. Under resting conditions, IκB binds to NF-κB and Bach1 binds to Maf recognition element (MARE), they form a new complex respectively, preventing NF-κB and Bach1 translocation from the cytoplasm to the nucleus 93. Once oxidative stress stimulates the complex, NF- κB dissociates from IκB, Bach1 dissociates from MARE. Both of them can activate the transcription of HO-1 18, 43.

## HO-1 and gynecological tumors

### Ovarian caner

In early stage of ovarian cancer (OC), patients lack specific manifestations, so early lesion is very easy to neglect. When patients show symptoms related to OC, they're usually at their advanced stage, tumor progresses rapidly, plus no effective treatment, therefore, the fatality rate of OC ranks first among gynecological malignancies 48.

Just as HO-1 is elevated in many tumors, the level of HO-1 in ovarian cancer is higher than that in normal ovarian tissues, which may be achieved by activating the VEGF. The VEGF/VEGFR signaling pathway is a key regulator of tumor angiogenesis, upregulating VEGFR2 can significantly increase the level of HO-1, which is a downstream target gene of Nrf2 8, 94.

Apatinib is a novel tyrosine kinase inhibitor, which can specifically target vascular endothelial growth factor receptor 2 (VEGFR2) and maintain it at a low level in OC cells, inhibiting the migration and proliferation of endothelial cells induced by VEGFR2 66. A study found that after Apatinib treatment, the levels of pro-apoptotic protein bax in OC cells increased, while the expression of p62 and anti-apoptotic protein Bcl-2 reduced 95. Light chain 3 (LC3) is an autophagy marker. When the cell undergoes autophagy, the cytoplasmic type (LC3-I) will decompose a small piece of peptide and transform into the membrane type (LC3-II). Therefore, the ratio of LC3-II/I can be used to evaluate the level of cell autophagy. In this experiment, the conversion of LC3-I to LC3-II also increased 9. This may be because Apatinib can act directly on OC cells, reducing tumor microvessel density, down-regulating the Nrf2/HO-1 pathway 93, 96, and promoting glutathione to generate ROS 97. In short, inhibiting Nrf-2/HO-1 pathway promotes ROS-dependent apoptosis and autophagy in OC cells 66, 93, 95, and plays an anti-tumor role 10.

EMT plays a key role in the growth and metastasis of tumor. HO-1 promotes the proliferation and migration of ovarian cancer cells by affecting EMT 98. Therefore, the level of HO-1 is closely related to its lymph node metastasis and FIGO stage. After treating OC tissue with heme inducer for 24 hours, vimentin (a mesenchymal marker), Zeb1 (a EMT transcription factor) and anti-apoptotic protein Bcl-2 were up-regulated; the levels of keratin and pro-apoptotic protein Bax decreased. The result is opposite after treating OC tissue with HO-1 inhibitor Znpp for 24 hours 9, suggesting that patients with high HO-1 expression have poorer prognosis and much lower overall survival.

In addition, endometriosis can lead to endometriosis-associated ovarian cancer (EAOC) 99, although this malignant transformation is relatively rare 48. Studies have shown that the number of M2 macrophages expressing HO-1 in EAOC is significantly reduced 100, suggesting that redox imbalance may participate in the malignant transformation of endometriosis 54. However, more research is still needed to confirm this theory.

### Cervical cancer

Cervical cancer (CC) is the most common female malignancy with a high incidence among 50-55 years-old women. However, in recent years, patients tend to be younger and younger 91. When squamous intraepithelial lesion (SIL) forms and continues to develop, it can exceed the basal layer, infiltrate the interstitial tissues and form invasive carcinoma.

Human papilloma virus (HPV) infection is the most important cause of CC. The oncoprotein produced by HPV may lead to autophagic dysfunction and the hinder viral clearance. Studies have found that HO-1 is overexpressed in HPV-infected tissues, especially in low-grade cervical intraepithelial neoplasia (CIN) lesions. X-linked inhibitor of apoptosis (XIAP) is an anti-apoptotic molecule and is considered to be a negative regulator of autophagy. By downregulating the expression of XIAP, HO-1 also increases the ratio of LC3-II/I 101.

On the other hand, HO-1 is a target gene of Nrf2, and its elevated level leads to the overexpression of Nrf2 in CC cells, which promotes the proliferation and invasion ability of SiHa cells 42, and makes tumor cells become insensitive to treatment 42, 91. Meanwhile, high levels of HO-1 are closely related to the clinical staging of cervical cancer, lymph node metastasis and poor prognosis.

Knockout of HO-1 can stimulate the inherent cellular response, promote autophagy response of CC cells, and downregulate the expression of anti-apoptotic proteins through anti-viral mechanisms. Hyperthermia is one of the adjuvant therapies for cervical cancer 17. A research showed that hyperthermia could down-regulate the expression of HO-1 in cervical cancer cells, reduce the viral load of HPV16 E6, and even destroy the existing physical state of HPV16. The combination of the two methods has a better effect than a single one 101.

### Endometrial cancer

Endometrial cancer (EC) usually occurs in elderly women, irregular vaginal bleeding after menopause is a typical symptom. EC can be divided into 2 types, type1 refers to endometrioid carcinoma, which accounts for 75%. As an estrogen-dependent type, it's closely associated with hyperplastic proliferation of the endometrial glands 102. Compared with normal tissues, endometrial hyperplasia (EH) is characterized by an increased ratio of endometrial glands to stroma by more than 1:1.Dysplasia of endometrium is even considered to be precancerous lesions, up to 50% patients will finally develop into EC 103, 104. A study carried by Fatma et al. has showed that increasing the expression of HO-1 can ameliorate the EH induced by estrogen,which maybe achieved by the suppression of inducible nitric oxide synthase (iNOS), p38, MAPK, and Ki67 43. However, in EC tissues, HO-1 is highly expressed. This is because EC often has irregular vaginal bleeding, which may be related to the massive release of heme. As is mentioned before, the degration porducts of heme are potentially toxic. Through mediating oxidative stress and inflammatory stimuli, large amount of heme in turn accelerates the occurrence and development of tumors 53. Overall, HO-1 shows completely different effects in EH tissues and in EC. However, articles on the role of HO-1 in EC are very limited and its mechanism is still unclear, which is worthy of further investigation.

## HO-1 and treatment insensitivity

Chemotherapy is a very important adjuvant treatment method after malignant tumor surgeries 89. Many commonly-used chemotherapy drugs can generate ROS to achieve the purpose of inducing cancer cell apoptosis 65, 96, 98. In normal situations, antioxidants can protect our body from oxidative stress damage; nevertheless, cancer cells may also activate antioxidant signals to fight against the damage caused by chemotherapy drugs, namely, cells develop drug resistance 105. Cis-platinum (CDDP) is one of the most widely used chemotherapy drugs 8. Recent reports indicated that its cytotoxic effect may be achieved by ROS-dependent apoptosis or DNA damage 17, 106. Other people believe that this may because chemotherapy-generated tumor cell debris hijack tumor-associated macrophages (TAMs). By promoting HO-1 expression and reducing M1-like polarization, tumor cells developed resistance to chemotherapy drugs. What's more, overexpression of HO-1 is often accompanied by an increase in multidrug resistance-related proteins (Mrp), which is an important reason for the difficulty in tumor treatment 67.

HO-1 plays a protective role in tumor cells has been widely recognized 107. Studies have found that HO-1 inhibitor ZnPPIX can improve sensitivity to chemotherapy of gastric cancer cell 12, 108, 109, other research showed that the inhibition rate of esophageal cancer cells was positively correlated with ZnPPIX concentration 109, 110. A study based on the treatment of acute myelogenous leukemia (AML) has found that through non‐covalently modified, lipid‐polymer hybrid nanoparticle loaded with HO1‐inhibitor tin mesoporphyrin (SnMP) can significantly improve the efficacy of daunorubicin and boost immune response 17, 111. Furthermore, a strong potential of blocking HO-1 for the treatment of hereditary leiomyomatosis and renal cell carcinoma (HLRCC) has already been verified 69. All these findings remind us that exploring the possibility of targeting or genetically or pharmacologically inhibiting HO-1 could make immunotherapy more effective 112, and HO-1 inhibitor may be used as a potential chemotherapeutic sensitizer in the near future 113.

## Conclusion and Prospective

Heme/HO system is one of the most important anti-oxidant mechanisms in our bodies. As a potential novel oncogene, HO-1 has received increasing levels of attention in recent years. On the one hand, HO-1 is highly expressed in a variety of gynecological malignancies, so the deviation of its dynamic trend from baseline could be used as a signal of disease alert and a predictor for the occurrence of tumors. On the other hand, for oncology patients with clinical manifestations and imaging evidence, high-level of HO-1 can also assist diagnosis. HO-1 level has certain relevance with prognosis and can be used as a potential indicator. Through inhibiting HO-1 directly or indirectly, HO-1 inhibitors can promote ROS-dependent autophagy and apoptosis. At present, HO-1 inhibitors have been used in clinical work and achieved certain efficacy.

However, the exact mechanism of HO-1 in gynecological tumors is still unclear. For one thing, HO-1 was proposed as a novel oncogene in gynecological malignancies not long ago, thus it has not yet been fully studied. For another, HO-1 seems to be tissue-specific. In normal tissues, it plays the role of anti-inflammation and anti-apoptosis, which indicates us to seek for non-stress HO-1 inducers for body protection. However, in cancer cells, HO-1 facilitates angiogenesis and tumor metastasis in turn. The contradiction makes the research on HO-1 very difficultly.

Anyway, regulating the expression of HO-1 may be a potential target of clinical treatment for patients with gynecological malignancies, although further studies are still needed to be done. Clarifing the exact mechanism of HO-1 in gyncological cancers may pave a new way for preventing the onset or progression of gyncological cancers.

## Figures and Tables

**Figure 2:**
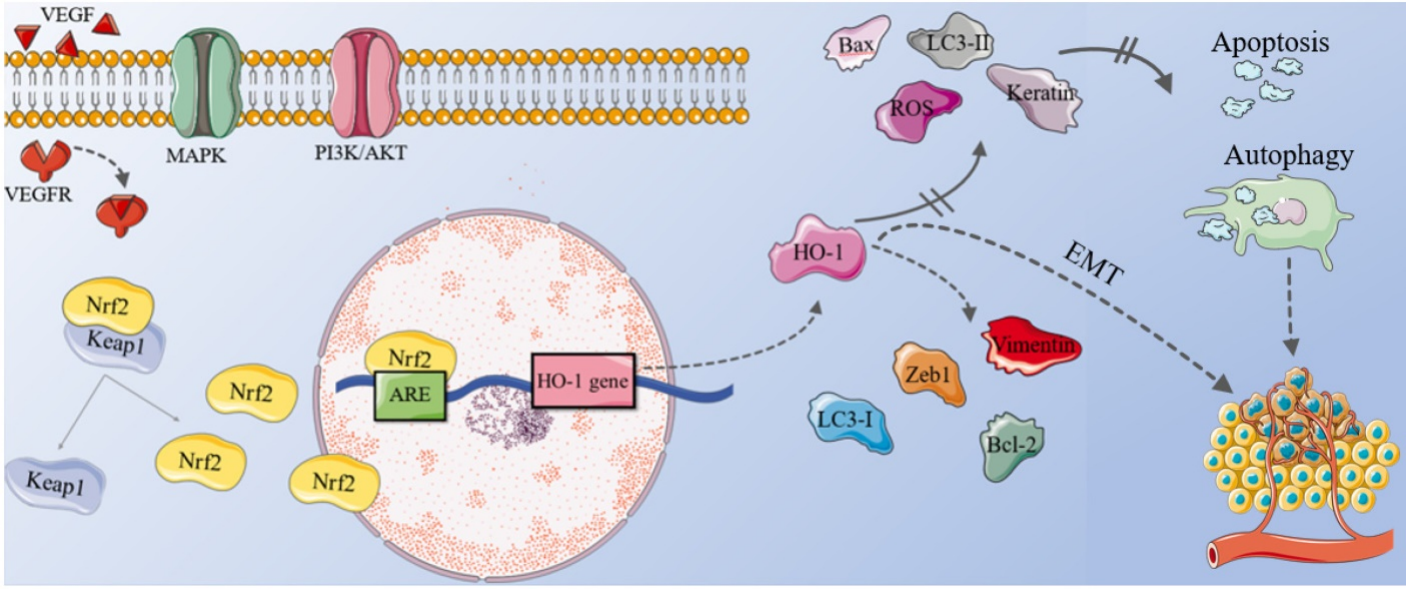
** The mechanism of HO-1 in ovarian cancer.** In OC cells, the combination of VEGF and VEGFR, plus the abnormal activation of MAPK and PI3K/ AKT signaling pathways can cause Nrf2 to dissociate from the Nrf2-Keap1 complex and increase the translocation of nucleus. Within the nucleus, Nrf2 binds to ARE, activating the transcription of its downstream gene HO-1. High levels of LC3-I, vimentin, Zeb1 and Bcl-2 can be observed in OC cells, while the level of LC3-II, keratin, Bax decrease. Therefore, up-regulating of the Nrf2/HO-1 pathway inhibits ROS-dependent apoptosis and autophagy in OC cells, increasing tumor microvessel density and promoting the growth and metastasis of OC. EMT also participate in this process collaboratively. OC: ovarian cancer; VEGF: vascular endothelial growth factor; VEGFR: VEGF receptor; Nrf2: nuclear factor E2-related factor 2; Keap1: kelch-like ECH-related protein 1; ARE: antioxidant-responsive element; LC3: light chain3; EMT: epithelial-mesenchymal transition.

**Table 1:** Comparison of three HO isozymes

	HO-1	HO-2	HO-3
**Molecular weight** **Inducibility** **Activity level**	32kD Stress-inducible High	36kD Constitutive High	Unknown Unknown Low
**Highly-Expressed localization**	Live, spleen, bone marrow, senescent erythrocytes	Brain, retina and testis	Widely distributed
**Similarity**	Degrade heme into biliverdin, carbon monoxide, ferrous ion and bilirubin		
